# Supplementary material for: Anisotropic atom motion on a row-wise antiferromagnetic surface
Source: Nat Commun. 2025 May 28;16:4942. doi: 10.1038/s41467-025-60086-9 (PMC12120023; doi:10.1038/s41467-025-60086-9)
Supplement: Supplementary file 1 — Supplementary Information [file 41467_2025_60086_MOESM1_ESM.pdf]

Supplemental Material:
Anisotropic atom motion on a row-wise antiferromagnetic surface

Felix Zahner,[1] Soumyajyoti Haldar,[2,*] Roland Wiesendanger,[1]
Stefan Heinze,[2,3] Kirsten von Bergmann,[1] and André Kubetzka[1,†]

[1]*Institute of Nanostructure and Solid State Physics (INF), University of Hamburg, Jungiusstraße 11, 20355 Hamburg, Germany*

[2]*Institute of Theoretical Physics and Astrophysics, University of Kiel, Leibnizstrasse 15, 24098 Kiel, Germany*

[3]*Kiel Nano, Surface, and Interface Science (KiNSIS), University of Kiel, 24118 Kiel, Germany*

(Dated: May 6, 2025)

I. SIMULATED STM/SP-STM IMAGES OF MN/RE(0001)

A comparison of simulated (non-magnetic) STM and SP-STM images with and without a shift of the pseudomorphic Mn layer in the row-wise AFM state is shown in Fig. S 1 for a small energy window above and below the Fermi level, respectively. The STM and SP-STM images are calculated based on the electronic structure from DFT using the Tersoff-Hamann model [1] and its extension to spin-polarized STM [2].

The simulated SP-STM images (Fig. S 1b,d,f,h) show the expected stripe period of the row-wise AFM state observed experimentally in Fig. 2a (main text). This magnetic contrast is the same for the unshifted and for the laterally shifted Mn layer. In the simulated STM images for a positive bias voltage, we find for the laterally shifted Mn layer (Fig. S 1e and Fig. 2d) also the experimentally observed stripes of atomic period shown in Fig. 2c. This stripe contrast is of electronic origin and it is absent in the simulated STM images of the unshifted Mn layer for both positive and negative bias voltages (Fig. S 1a,c). This supports that the lateral shift of the Mn layer predicted by our DFT total energy calculations occurs in the experiment.

* haldar@physik.uni-kiel.de
† kubetzka@physnet.uni-hamburg.de

II. CO ELECTRIC DIPOLE AND VOLTAGE POLARITY

Concerning voltage polarity, the data of Fig. 3f shows a trend to larger kicking distances for negative polarity, a much weaker effect than observed for other systems [3–5]. In further experiments with $U_\mathrm{P} = \pm 500$ mV we found no statistically significant effect of polarity on travel distance, indicating that the local electric field does not dominate the Co atom movement. In addition, other effects can cause a voltage polarity dependence, for instance an asymmetric density of states with respect to the Fermi level. Indeed, the vacuum density of states of Mn/Re(0001) is about four times higher 200 meV below compared to 200 meV above the Fermi level (see Fig. S9 in Ref. 6). This leads to a higher current flowing at pulses of $U_\mathrm{P} = -200$ mV compared to $U_\mathrm{P} = +200$ mV, which might explain the polarity trend observed in Fig. 3f.

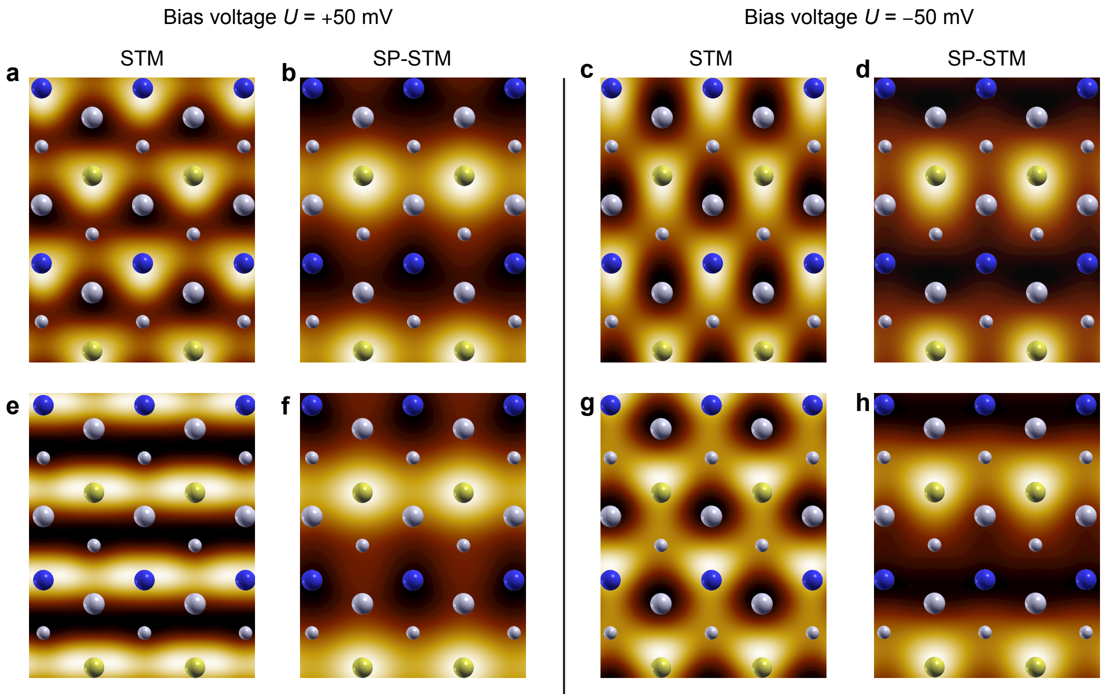

Fig. S 1 STM simulations for Mn/Re(0001) at a bias voltage of $U = \pm 50$ mV. Comparison of simulated STM and SP-STM images of fcc-Mn/Re(0001) in the row-wise AFM state based on DFT calculations at a bias voltage of ±50 meV and a distance of 3 Å above the surface without and with a lateral shift of the Mn monolayer. **a-d** Mn atoms are at perfect fcc positions. **e-h** Mn atom rows shifted from the perfect fcc sites into the energetically favorable relaxed positions. Yellow and blue spheres represent Mn atoms of opposite spin directions, while white spheres denote Re atoms. The big and small white spheres denote surface and subsurface Re atoms. SP-STM images assume a tip spin-polarization of 0.5.

To further investigate the impact of the tip's local electric field onto the atom movement we have calculated the Co electric dipole using the Bader charge analysis [7]. It turns out that the value is sizable with $\mu = 0.61$ eÅ or 2.9 D. For comparison, H_2O has an electric dipole of $\mu = 1.85$ D. When a charge-neutral Co atom is placed on the Mn layer, electrons are transferred from the surface toward the Co atom, producing an electric dipole pointing down. Neglecting work function differences between tip and sample, at positive (negative) voltage the tip's electric field points up (down), decaying with lateral distance from the tip position. Thus, a repulsive (attractive) force should act on the Co atom at positive (negative) voltage. This means that the weak polarity trend observed in Fig. 3f cannot be explained by the tip's electric field. Thus, despite the sizable Co dipole, electric fields do not play a role in the experiments.

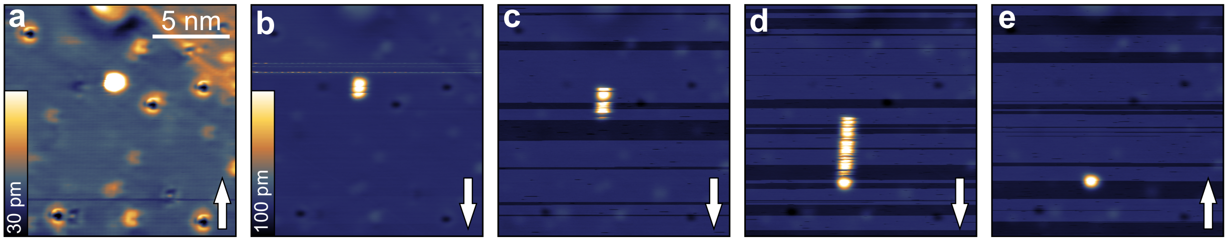

Fig. S 2 Scanning across an Ir atom at increasing tunnel voltage. a Resting Ir atom on fcc-Mn/Re(0001), with the ↑↑-rows running vertically, imaged at $U = +100\,\mathrm{mV}$. The slow tip scanning direction is indicated by a white arrow. **b** Ir atom moving increasing distances downward (along the ↑↑-rows) while imaged at $U = +1\,\mathrm{V}$, **c** $U = +2\,\mathrm{V}$, and **d** $U = +2.5\,\mathrm{V}$. **e** Ir atom resting at a native defect imaged at $U = +2.5\,\mathrm{V}$. This data series indicates that one-dimensional movement along the ↑↑-rows can be expected for Ir atoms, with barriers even higher than for Rh and Co atoms. All images were taken at $I = 50\,\mathrm{nA}$ and $T = 4.2\,\mathrm{K}$.

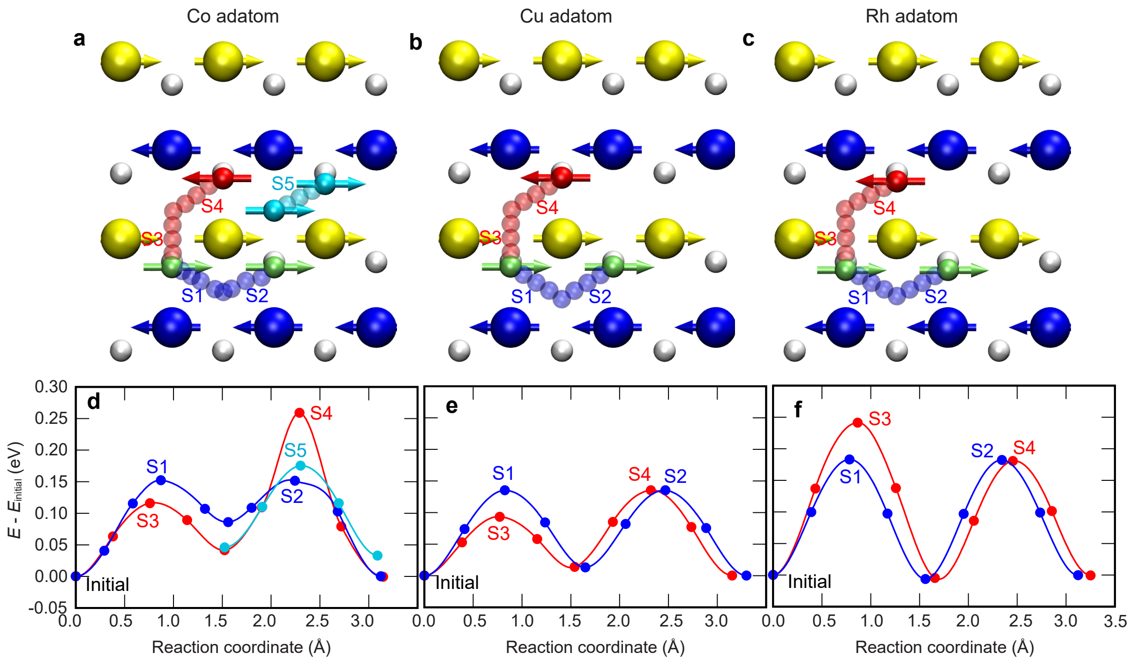

Fig. S 3 Minimum energy paths. **a-c** Minimum energy paths and the positions of saddle points along the paths for Co, Cu, and Rh adatoms on Mn/Re(0001), respectively. **d-f** Energy barrier plots along the paths shown above together with the saddle point positions for Co, Cu, and Rh adatom, respectively.

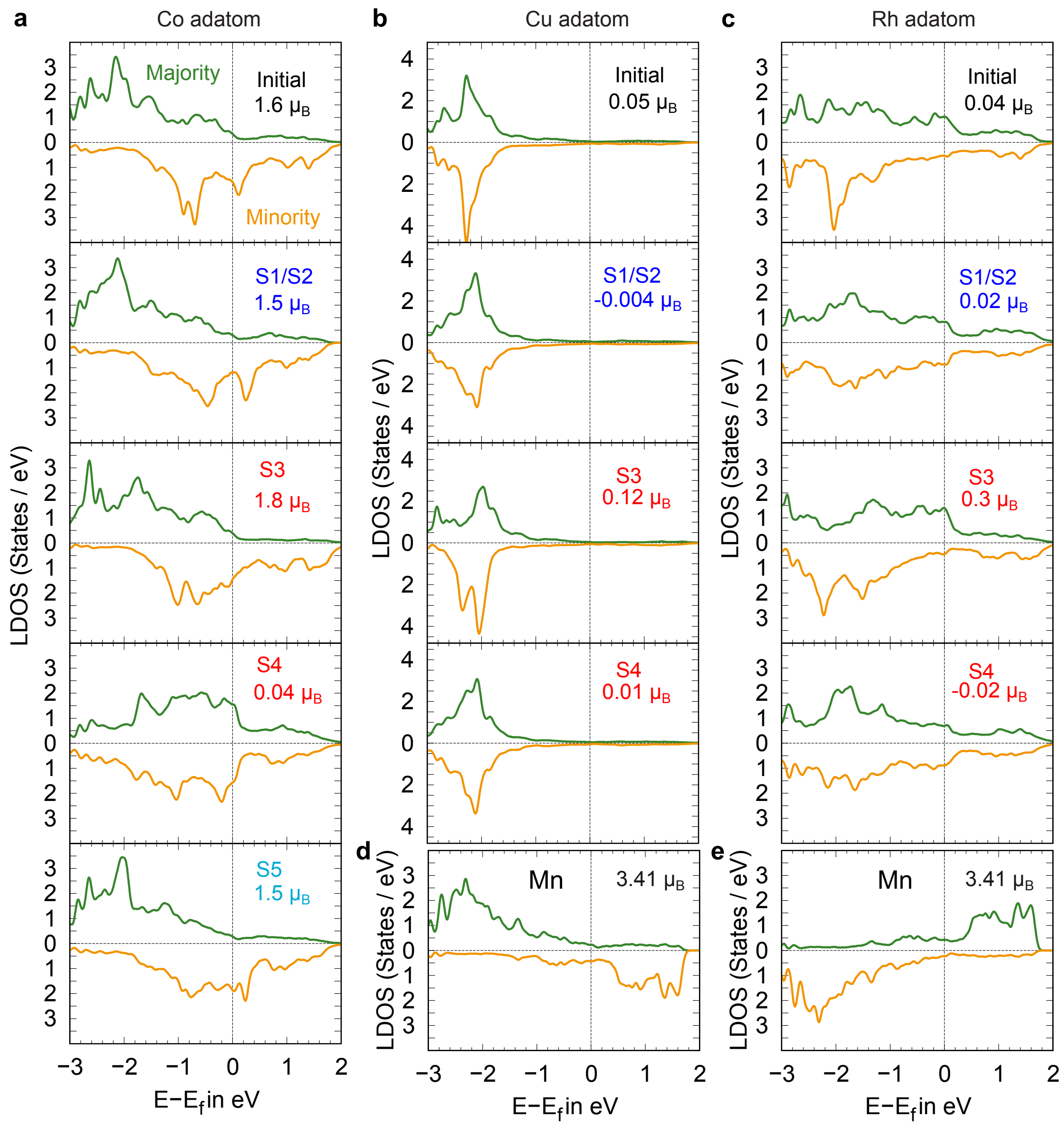

Fig. S 4 Local density of states highlighting different spin-dependent hybridization of the adatoms with the Mn surface atoms. a-c Comparison of the spin-resolved local density of states (LDOS) between the initial state and the different saddle points for the Co, Cu, and Rh adatom, respectively (cf. Fig. S2 for saddle points). Spin-up (majority) and spin-down (minority) channel LDOS are shown by green and orange curves along the positive and negative y-axis, respectively. The values in the panels indicate the magnetic moment of the adatom in the unit of μ_B. **d,e** LDOS of the two Mn surface atoms with opposite magnetic moments of the unperturbed Mn layer on Re(0001), i.e. without adatom adsorption.

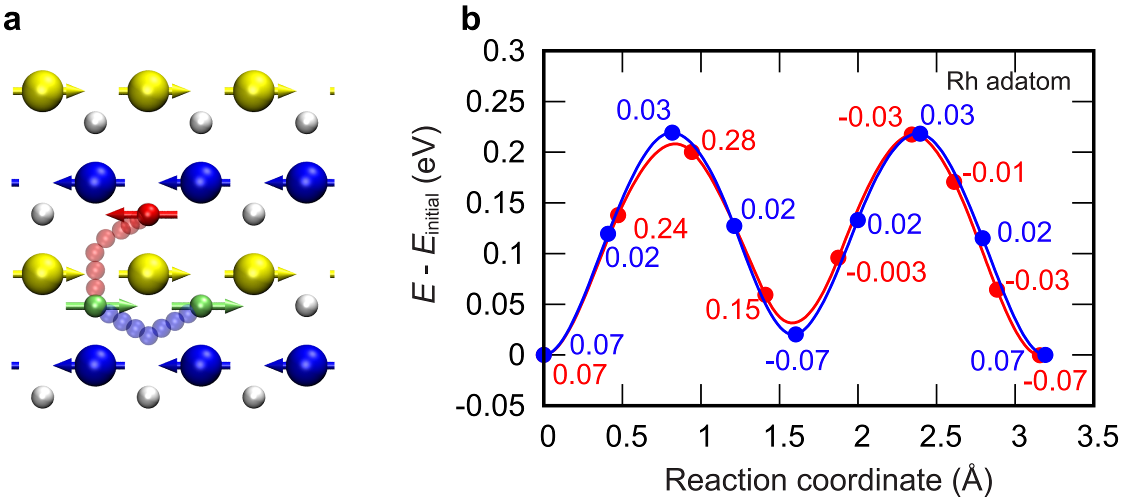

Fig. S 5 Minimum energy path of Rh on the laterally unrelaxed Mn layer. **a** Minimum energy path and the positions of saddle points along the paths for Rh adatoms on Mn/Re(0001) where the Mn atoms are not laterally shifted, i.e. the magnetism-induced lateral shift of the Mn layer has been neglected in these DFT calculations. **b** Energy barrier plots along the paths shown above together with the saddle point positions for Rh adatom where Mn atoms are not laterally shifted.

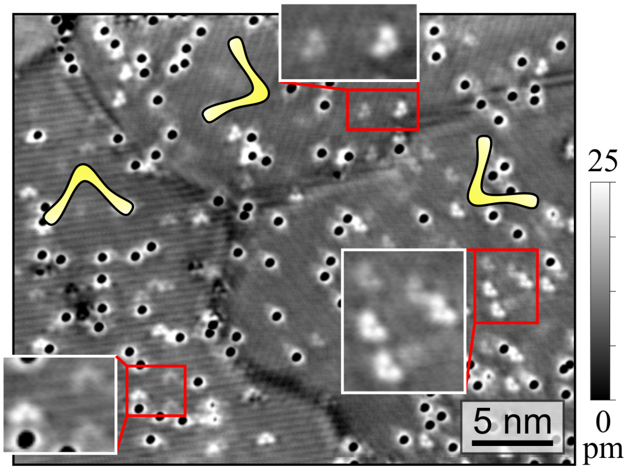

Fig. S 6 Correlation of row-wise AFM state and defect shape. Intersection of three rotational domains, two of them showing an almost vanishing spin contrast ($U = -30$ mV, $I = 7$ nA). Some defect types display a broken C_3 symmetry, correlated with the direction of the ↑↑-rows, compare zoom-ins and yellow boomerangs. This correlation allows to determine the orientation of row-wise AFM domains in non-magnetic STM measurements.

[1] J. Tersoff and D. R. Hamann, Theory and Application for the Scanning Tunneling Microscope, Phys. Rev. Lett. **50**, 1998 (1983).

[2] D. Wortmann, S. Heinze, P. Kurz, G. Bihlmayer, and S. Blügel, Resolving Complex Atomic-Scale Spin Structures by Spin-Polarized Scanning Tunneling Microscopy, Phys. Rev. Lett. **86**, 4132 (2001).

[3] L. J. Whitman, J. A. Stroscio, R. A. Dragoset, and R. J. Celotta, Manipulation of Adsorbed Atoms and Creation of New Structures on Room-Temperature Surfaces with a Scanning Tunneling Microscope, Science **251**, 1206 (1991).

[4] J. A. Stroscio and D. M. Eigler, Atomic and Molecular Manipulation with the Scanning Tunneling Microscope, Science **254**, 1319 (1991).

[5] D. Civita, M. Kolmer, G. J. Simpson, A.-P. Li, S. Hecht, and L. Grill, Control of long-distance motion of single molecules on a surface, Science **370**, 957 (2020).

[6] J. Spethmann, S. Meyer, K. von Bergmann, R. Wiesendanger, S. Heinze, and A. Kubetzka, Discovery of Magnetic Single- and Triple-**q** States in Mn/Re(0001), Phys. Rev. Lett. **124**, 227203 (2020).

[7] W. Tang, E. Sanville, and G. Henkelman, A grid-based Bader analysis algorithm without lattice bias, J. Phys.: Condens. Matter **21**, 084204 (2009).
