# Supplementary material for: Anisotropic atom motion on a row-wise antiferromagnetic surface
Source: Nat Commun. 2025 May 28;16:4942. doi: 10.1038/s41467-025-60086-9 (PMC12120023; doi:10.1038/s41467-025-60086-9)
Supplement: Supplementary file 2 — Description Of Additional Supplementary File [file 41467_2025_60086_MOESM2_ESM.pdf]

Description of Additional supplementary file

Supplementary Movie 1

Kicking of single Co atoms on the row-wise antiferromagnetic state of Mn/Re(0001). This video is associated with Fig. 3 of the main text. Left: STM image of two rotational domains and a domain wall (DW); domain orientations are indicated. Right: 4 STM frames alternating between images taken before and after a voltage pulse of U = +200 mV above the atoms marked with crosses.

Supplementary Movie 2

Kicking of single Rh atoms on the row-wise antiferromagnetic state of Mn/Re(0001). This video is associated with Fig. 4 of the main text. Left: STM image of two rotational domains and a domain wall (DW); domain orientations are indicated. Right: 4 STM frames alternating between images taken before and after a voltage pulse of U = +1 V above the atoms marked with crosses.
